# Gastric Inhibitory Polypeptide Receptor Methylation in Newly Diagnosed, Drug-Naïve Patients with Type 2 Diabetes: A Case-Control Study

**DOI:** 10.1371/journal.pone.0075474

**Published:** 2013-09-23

**Authors:** Silvia Canivell, Elena G. Ruano, Antoni Sisó-Almirall, Belchin Kostov, Luis González-de Paz, Eduardo Fernandez-Rebollo, Felicia Hanzu, Marcelina Párrizas, Anna Novials, Ramon Gomis

**Affiliations:** 1 Department of Endocrinology and Nutrition, Hospital Clinic-Institut d’Investigacions Biomèdiques August Pi i Sunyer, Barcelona, Spain; 2 Les Corts Primary Health Care Centre, Transverse group for research in primary care-Institut d’Investigacions Biomèdiques August Pi i Sunyer, Barcelona, Spain; 3 Diabetes and Obesity Laboratory-Institut d’Investigacions Biomèdiques August Pi i Sunyer, Barcelona, Spain; 4 Spanish Biomedical Research Centre in Diabetes and Associated Metabolic Disorders, Barcelona, Spain; 5 University of Barcelona, Barcelona, Spain; University of Lancaster, United Kingdom

## Abstract

GIP action in type 2 diabetic (T2D) patients is altered. We hypothesized that methylation changes could be present in GIP receptor of T2D patients. This study aimed to assess the differences in DNA methylation profile of *GIPR* promoter between T2D patients and age- and Body Mass Index (BMI)-matched controls. We included 93 T2D patients (cases) that were uniquely on diet (without any anti-diabetic pharmacological treatment). We matched one control (with oral glucose tolerance test negative, non diabetic), by age and BMI, for every case. Cytokines and hormones were determined by ELISA. DNA was extracted from whole blood and DNA methylation was assessed using the Sequenom EpiTYPER system. Our results showed that T2D patients were more insulin resistant and had a poorer β cell function than their controls. Fasting adiponectin was lower in T2D patients as compared to controls (7.0±3.8 µgr/mL vs. 10.0±4.2 µgr/mL). Levels of IL 12 in serum were almost double in T2D patients (52.8±58.3 pg/mL vs. 29.7±37.4 pg/mL). We found that *GIPR* promoter was hypomethylated in T2D patients as compared to controls. In addition, HOMA-IR and fasting glucose correlated negatively with mean methylation of *GIPR* promoter, especially in T2D patients. This case-control study confirms that newly diagnosed, drug-naïve T2D patients are more insulin resistant and have worse β cell function than age- and BMI-matched controls, which is partly related to changes in the insulin-sensitizing metabolites (adiponectin), in the proinflammatory profile (IL12) and we suggest in the methylation pattern of *GIPR*. Our study provides novel findings on *GIPR* promoter methylation profile which may improve our ability to understand type 2 diabetes pathogenesis.

## Introduction

Twin cohort studies have shown that shared genetic factors can only explain a fraction of the differences in incident type 2 diabetes (T2D) [1]. Behavioral (sedentary lifestyle, westernized food patterns) and environmental factors (organic pesticides, chemical exposures, and air pollutants) contribute to the development of T2D [2]. Moreover, inflammation induces inhibition of the insulin signalling pathway which can lead to insulin resistance and T2D. Recently, it has been proposed that epigenetic mechanisms could be involved in the complex interplay between genes and the environment [3]. Indeed, a recent study showed the presence of an epigenetic dysregulation in pancreatic islets from T2D patients [4]. Briefly, they found differences in DNA methylation profiles in several promoter regions in islets from T2D patients [4]. DNA methylation is the best studied epigenetic modification and influences transcripcional regulation [5]. DNA methylation is a reversible process that can be modulated by both stochastic and environmental stimuli [6]. On the other hand, *GIPR* gene codifies for the receptor of the incretin GIP, a gastrointestinal hormone that stimulates insulin response after an oral glucose challenge. In T2D patients, GIP action is reduced, whereas its secretion does not seem to be altered. There is increasing evidence supporting an important role for *GIPR* as a candidate for mediating insulin secretion after oral glucose challenge [7]. We speculated that *GIPR* gene could be affected by alterations in DNA methylation in T2D patients, which could explain the dysregulation of GIP action in T2D patients [8], [9]. As DNA methylation occurs principally in the upstream regulatory regions of the genes [10], we concentrated on the promoter of *GIPR*. A previous study has shown that T2D-related methylation may be reflected in accessible tissues such as peripheral blood [11].

The principal aim of this study was to compare the pattern of DNA methylation on *GIPR* promoter between T2D patients and age- and Body Mass Index (BMI)-matched controls. The secondary aims were to compare the metabolic and cytokine profiles between T2D patients and matched controls.

## Materials and Methods

### Ethics Statement

This study was approved by the ethics committees of the Hospital Clínic and complies with all laws and international ethics guidelines outlined in the Declaration of Helsinki. All human subjects provided written, informed consent.

### Study Design and Subjects Included

We conducted a case-control study where cases were defined as patients suffering from T2D that were treated only by diet. Eligibility criteria for cases were the following: clinical diagnosis of T2D between December 2010 until December 2011, adequate glycemic control after a period of minimum six months of low-carbohydrate diet and lifestyle interventions, no pharmacological therapy for T2D needed to achieve the glycemic control. Diagnosis of T2D was done following ADA recommendations [12], by either a random elevated fasting glucose value (confirmed twice) and/or by performing an oral glucose tolerance test (OGTT). In case oral medication was needed for optimal glycemic control, those patients were excluded from the study. Cases and controls were recruited from the same primary health center. Eligibility criteria for controls were as follows: a negative OGTT at recruitment, no previous diagnosis of T2D or prediabetes, no chronic treatment with oral steroids. Controls were frequency matched on age and BMI to cases. Metabolic profile, cytokine profile and DNA methylation of *GIPR* promoter profile in peripheral blood DNA were studied for all subjects (93 cases and 93 controls).

### Metabolic Assessments

All subjects were examined by anthropometric measurements and had fasting metabolic assessments at recruitment. These assessments included fasting glucose, fasting insulin, fasting leptin, fasting adiponectin, cytokines, glycohemoglobin A1 (HbA1) (only for the type 2 diabetic patients), HOMA-IR and HOMA-B. HOMA-IR was calculated as follows: HOMA-IR = (FPI × FPG)/22.5 [13]; homeostasis β-cell function (HOMA-B) = (20 × FPI)/(FPG − 3.5), where FPI is the fasting plasma insulin concentration (mU/l) and FPG is fasting plasma glucose (mmol/l) [14].

### Hormone and Cytokine Measurements

Adiponectin, leptin and insulin were quantified from serum samples by ELISA (Mercodia), according to the manufacturer’s instructions. Cytokines were measured from serum samples using CBA Human Inflammatory Cytokines kit (BD Bioscience), following the manufactureŕs instructions. Two-color flow cytometric analysis was performed using LSRFortessa (BD bioscience). Data were acquired and analyzed using FACS Diva and FCAP Array 1.01 Software. Hormone and cytokine measurements were performed at the Diabetes and Obesity Laboratory-IDIBAPS; Barcelona, Spain.

### DNA Methylation Analysis

Whole blood samples were stored in the Biobank Hospital Clínic-IDIBAPS; Barcelona, Spain. Genomic DNA was extracted from whole blood for all the subjects studied using standards procedures from the Biobank. Sequenom's MassARRAY platform was used to perform quantitative methylation analysis [15]. This system utilizes MALDI-TOF mass spectrometry in combination with RNA base-specific cleavage (MassCLEAVE). A detectable pattern is then analyzed for methylation status. PCR primers for the amplification of the *GIPR* promoter gene were designed using *Epidesigner* (See Appendix S1).

### Statistical Analysis

Descriptive data are presented as the mean and standard deviation (SD) for continuous outcomes, or number and percentage (%) for categorical outcomes. The methylation values (in %), cytokines, HOMA-IR, HOMA-B, insulin, leptin and adiponectin were compared using non-parametric Mann-Whitney U test, because normality and equality of variance could not be assumed. Student’s *t* test was used for the comparison of the rest of continuous outcomes and Chi-square test for categorical outcomes. Correlation between methylation at all thirteen CpG sites was high (*P* = 0.002), therefore a mean of *GIPR* promoter methylation was generated. Spearman’s rank correlation coefficient was used to assess correlation between mean *GIPR* promoter methylation and the different covariates (waist circumference, fasting glucose, fasting insulin, fasting adiponectin, fasting leptin, HOMA-IR, HOMA-B, cytokines). Linear regression was used to study the association between the mean *GIPR* promoter methylation (independent variable) and the covariates (dependent variables) that presented a significant correlation in the Spearman analysis, after adjustment for diabetis status (i.e, being case or control), sex, age and BMI. Mean *GIPR* promoter methylation was log-transformed for the regression analysis. Subgroup analyses (i.e, by disease status) were done for the variables that remained significant after the adjustment. Overall R^2^ values for the models give the combined contribution of log-transformed mean *GIPR* promoter methylation, sex, age, BMI and diabetes status to the variability in dependent variables. Bonferroni correction was used for multiple comparisons. All significance tests were 2-tailed and values of p<0.05 were considered significant. All analyses were conducted using the statistical software package Stata version 11.

## Results

### Metabolic and Cytokine Profile of Type 2 Diabetic Patients and Controls

Baseline characteristics of the patients included in the study are summarized in Table 1. T2D patients had a higher waist circumference as compared to controls (mean waist values of 102.7±9.5 cm vs. 97.9±8.0 cm, *P*<0.01). Fasting adiponectin was lower in cases as compared to controls (mean values of 7.0±3.8 µgr/mL vs. 10.0±4.2 µgr/mL, *P*<0.0001). HOMA-IR was higher in cases (2.6±1.5 vs. 1.8±0.7 in controls, *P*<0.0001). HOMA-B was higher in controls as compared to T2D patients (113.6±510.6 vs. 75.7±51.1 in type 2 diabetic patients, *P*<0.0001). From the cytokines analyzed, significant differences were found for IL 10 (4.1±3.0 pg/mL in cases vs. 5.2±3.7 pg/mL in controls, *P*<0.05) and IL 12 (52.8±58.3 pg/mL in cases vs. 29.7±37.4 pg/mL in controls, *P*<0.0001). No differences were found between cases and controls in the routine laboratory measures (blood cell count, hepatic profile, lipid profile, renal function, data not shown).

**Table 1:** Demographic and metabolic characteristics of type 2 diabetic patients and age- and BMI-matched controls.

Variable*	Type 2 diabetic patients (n = 93)	Controls (n = 93)	P Value†
**Demographic characteristics**			
Age, yr	69.1±9.2	66.6±11.7	Matching variable
BMI, kg/m^2^	29.2±3.7	28.8±2.5	Matching variable
Waist circumference, cm	102.7±9.5	97.9±8.0	**<0.01**
Male sex, (%)	66.7	53.8	0.07
**Laboratory values**			
Fasting glucose, (mmol/L)	6.4±1.2	4.6±0.4	**<0.0001**
Glycated hemoglobin, (%)	5·8±0.6	–	
Fasting insulin, (pmol/L)	55.6±28.6	52.4±21.1	0.39
HOMA-IR ‡	2.6±1.5	1.8±0.7	**<0**.**0001**
HOMA-B §	75.7±51.1	113.6±510.6	**<0**.**0001**
Fasting leptin, (ng/mL)	18.0±16.7	25.4±26.8	0.07
Fasting adiponectin, (µg/mL)	7.0±3.8	10.0±4.2	**<0**.**0001**

* Values shown are means ±SD, unless otherwise indicated.

† P values were calculated with the t test for quantitative variables or Chi-square test for categorical ones, except for HOMA-IR, HOMA-B, fasting insulin, fasting leptin and fasting adiponectin, where non-parametric Mann-Whitney U test was applied.

‡ HOMA-IR was calculated as [Insulin mUI/l x Glycemia: (mmol/l)/22.5].

§ HOMA-B was calculated as (20 × FPI)/(FPG − 3.5), where FPI is the fasting plasma insulin concentration (mU/l) and FPG is fasting plasma glucose (mmol/l).

### Quantitative DNA Methylation Analysis in Peripheral Blood of *GIPR* Promoter in Type 2 Diabetic Patients and Controls

**Figure 1:**
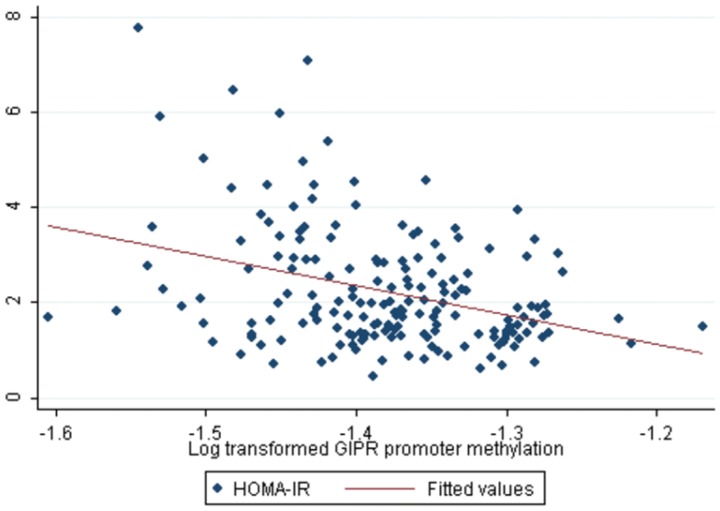
Correlation between average *GIPR* promoter methylation from peripheral blood DNA and insulin resistance. Log-transformed average *GIPR* promoter methylation is shown as the independent variable. HOMA-IR was used as a marker of insulin resistance. Spearman ‘s correlation r = −0.29, *P* = 0.0001. Adjusted *P*<0.01 (diabetes status, age, BMI and gender).

**Figure 2:**
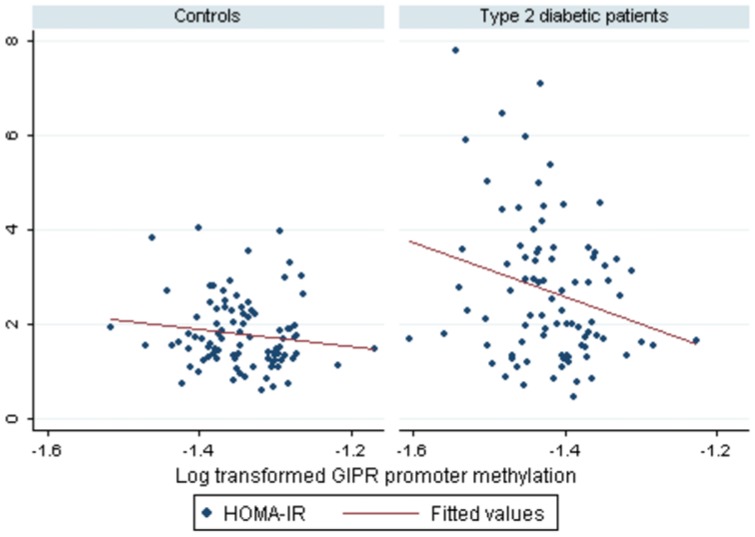
Correlation between average *GIPR* promoter methylation from peripheral blood DNA and insulin resistance, by subgroups (Type 2 diabetic patients and controls). Log-transformed average *GIPR* promoter methylation is shown as the independent variable. HOMA-IR was used as a marker of insulin resistance. *P* = 0.06, adjusted for age, BMI and gender in controls (n = 93). *P*<0.05, adjusted for age, BMI and gender in patients with Type 2 diabetes (n = 93).

Methylation levels in DNA from whole blood of 186 subjects were obtained for 13 CpG sites covering 1,000 bp upstream of the first exon of the human *GIPR* gene. The heatmap showing the values of methylation (%) for each CpG site analyzed did not reveal a clearly distinct pattern of methylation between T2D patients and controls (figure not shown); however some significant differences were found. Indeed, 9 out of 13 CpG sites studied (69%) showed significant differences between T2D patients and controls. There was a trend towards an hypomethylation in T2D patients as compared to controls (See Table S1). In fact, mean *GIPR* promoter methylation was lower in T2D patients as compared to controls (24.3±1.6 in cases vs. 26.2±1.5 in controls, *P*<0.0001). Mean methylation of *GIPR* promoter was correlated with waist circumference (r = −0.26, *P*<0.01), fasting glucose (r = −0.50, *P*<0.0001), HOMA-IR (r = −0.29, *P<*0.001), HOMA-B (r = 0.28, *P*<0.001), fasting adiponectin (r = 0.23, *P*<0.01) and IL-12 (r = −0.22, *P*<0.01) (see Table 2). After adjustment, increased *GIPR* promoter methylation was associated with decreasing fasting glucose [−2.4 (−4.5 to −0.2), *P*<0.05] and decreasing HOMA-IR [−4.6 (−7.5 to −1.8) *P*<0.01] (see Figure 1). Hence, following a 10% increase in log-transformed *GIPR* promoter methylation, fasting glucose and HOMA-IR decrease by 0.24 mmol/L and 0.46 units, respectively (see Table 2). The combined contribution of *GIPR* promoter methylation and diabetes status, age, sex and BMI to the variability in HOMA-IR was up to 23% and up to 53% regarding fasting glucose (see Table 2). Separate analyses of T2D patients and controls showed that the significant inverse correlation between mean *GIPR* methylation and HOMA-IR was mostly present in T2D patients (*P*<0.05) and not in controls (*P* = 0.06) (see Figure 2). Regarding fasting glucose, the relationship remained significant also uniquely for T2D patients (*P*<0.05), and not in controls (*P* = 0.80).

**Table 2:** Results of correlation analysis between *GIPR* promoter methylation and the listed dependent variables.

Outcome variable	Spearman’s correlation	Unadjusted p-value	Adjusted p-value*	R^2^ (%)†
**Waist circumference**	−0.26	**<0.01**	0.59. β = −5.2 (−24.4 to 13.9)	44.8
**Fasting glucose**	−0.50	**<0.0001**	**<0.05. β = **−**2.4 (**−**4.5 to** −**0.2)**	52.9
**Fasting insulin**	−0.12	0.10		
**HOMA-IR**	−0.29	**<0.001**	**<0.01. β = **−**4.6 (**−**7.5 to** −**1.8)**	22.7
**HOMA-B**	0.28	**<0.001**	0.53. β = −0.5 (−1.9 to 1.0)	39.4
**Fasting leptin**	−0·05	0·49		
**Fasting adiponectin**	0.23	**<0.01**	0.28. β = 5.1 (−4.1 to 14.2)	23.8
**IL-1B**	−0.02	0.75		
**IL-8**	−0.03	0.72		
**IL-6**	0.08	0.32		
**IL-10**	0.06	0.43		
**IL-12**	−0.22	**<0.01**	0.55. β = −37.7 (−161.6 to 86.1)	6.1
**TNFα**	0.03	0.65		

* Adjustment for age, BMI, sex and diabetes status by creating linear regression analyses between log-transformed GIPR promoter methylation and the dependent variables that presented a significant correlation in Spearman’s analysis. Regression coefficients and corresponding 95% CIs are shown.

† R^2^ reflects the variance (%) in outcome measures accounted for age, BMI, sex, diabetes status and GIPR promoter methylation.

## Discussion

The leading cause of T2D is thought to be an impaired β cell function [16] which depends on a complex interplay of genetic predisposition and environmental factors, such as obesity, inactivity and aging. In this sense, we aimed to compare, given a similar environment (defined as similar age and similar degree of obesity), which were the factors associated with the apparition of T2D. Therefore, we compared the metabolic and cytokine profile between 93 newly diagnosed T2D patients and 93 age- and BMI-matched controls. In addition, we also performed the first DNA methylation profiling of human peripheral blood covering the promoter of glucose-associated gene *GIPR* in T2D patients and controls.

T2D patients and controls were similar in age and BMI to control for any confounder effect of age and obesity on the results. Moreover, none of the T2D patients were on any pharmacological therapy for diabetes. Thus, no confounding effect of antidiabetic drugs or insulin therapy was possible, either. T2D patients had their clinical diagnosis of T2D recently and were in optimal glycemic control. Hence, no potential influence of hyperglycemia on the methylation pattern was possibe, or, if any, was low. Results showed that T2D patients were more insulin-resistant than controls, since they presented higher values of HOMA-IR. In concordance to this, T2D patients had a higher waist circumference as compared to controls. Large waist circumference is one component used for the diagnosis of the metabolic syndrome. Insulin resistance is associated with metabolic syndrome too [17]. Basically, in spite of the fact that T2D patients and controls had a similar grade of obesity, T2D patients presented a differential body fat distribution (particularly centralized obesity). This correlates with a differential adipokines secretion which might lead to a higher degree of insulin resistance in T2D patients. In contrast, and as expected, β cell function was already impaired in T2D patients as compared to controls (HOMA-B was significantly lower in T2D patients as compared to controls). These data illustrates the fact that impairment of β cell function is worse in T2D patients compared to age- and BMI- matched controls. These results are in concordance with the existing literature [18]. In addition, we found that T2D patients had lower adiponectin levels in serum than controls. Epidemiological studies have shown that higher adiponectin levels in serum are associated with a lower risk of T2D_ENREF_19. Moreover, adiponectin has been proposed as a strong biochemical predictor of T2D [19]. Adiponectin is exclusively and abundantly expressed in white adipose tissue and has been shown to have insulin-sensitizing and anti-inflammatory properties [20]. In fact, in our study, we found that fasting adiponectin had a negative correlation with HOMA-IR (Spearman correlation coefficient r = −0.28, *P*<0.0001) and a positive correlation with HOMA-B (r = 0.19, *P*<0.01), which supports the insulin-sensitizing properties of adiponectin.

On the other hand, lower levels of the anti-inflammatory IL-10 were found in T2D patients, which is consistent with previous research that showed that low levels of IL 10 are associated with T2D [21]. IL-12 serum levels were almost double in T2D patients than in controls. A recent study showed that elevated serum IL-12 was present at the onset of T2D, and that further increases in IL-12 correlated with endothelial dysfunction and cardiovascular disease progression [22]. In addition, it has also been showed that IL-12 might have a role in β cell dysfunction [23]. Overall, the first part of our research demonstrate that T2D patients have an impaired β cell function and are more insulin resistant than age- and BMI-matched controls. These differences in β cell function and insulin resistance are related to differences in adipokines and inflammatory metabolites, which might be the underlying mechanisms that lead to overt T2D [24].

Next, we performed a DNA methylation profiling of *GIPR* promoter in DNA from peripheral blood and we sought for associations of methylation with blood-and T2D-based biomarkers. We found that *GIPR* promoter was hypomethylated in T2D patients as compared to controls. These results are consistent with a recent study which showed that hypomethylation in specific genomic regions in peripheral blood DNA was associated with T2D [11]. However, their analysis did not cover the genomic region we studied. To our knowledge, *GIPR* promoter methylation analysis in peripheral blood DNA between T2D patients and age- and BMI-matched controls has not been done before. There is great interest to perform methylation profiling in peripheral blood to find methylation disease-related associations since specific methylated regions could be used as potent biomarkers [25]. However, to understand how these methylated regions have a mechanistic role in the development of the disease of interest, the methylation analysis should focus in the target-tissues of the genes studied. *GIPR*, or gastric inhibitory polypeptide receptor, gene is expressed in various tissues, including β cells, adipose tissue, and brain [26]. It has been shown that *GIPR* expression is down-regulated in pancreatic tissue of T2D patients [27]. Here, we found that methylation of *GIPR* promoter in blood was negatively correlated with a surrogate marker of insulin resistance (HOMA-IR) and fasting glucose. In other words, decreased methylation in this promoter is associated with higher insulin resistance and higher fasting glucose. The subgroup analysis showed that this association was mostly relevant for T2D patients. The mechanisms underlying this association remain unknown and were not the purpose of the current research. On the other hand, methylation of *GIPR* promoter was not associated with HOMA-B. It has been shown that *GIPR* is involved in obesity and insulin resistance [28]. Recently, GIP was proposed as having a role in inflammation and insulin resistance by modulating the expression of osteopontin in adipose tissue [29]. Moreover, carriers of *GIPR* rs10423928 A-allele showed better insulin sensitivity [29]. The possible DNA methylation contribution to these effects has not been studied yet and warrants further study. Methylation patterns are thought to be tissue-specific [4], [5], thus we might not extrapolate the methylation pattern found in blood to the methylation pattern present in adipose tissue. Further research is needed to define the role of methylation changes in *GIPR* promoter in adipose tissue and their potential impact on insulin resistance.

The strength of our research is that we have demonstrated that newly diagnosed and drug-naïve T2D patients have differences in specific hormones (adiponectin) and proinflammatory metabolites (especially IL 12) as compared to age- and BMI-matched controls. We also found that *GIPR* promoter was hypomethylated in T2D patients as compared to controls, as well as, new correlations between insulin resistance, fasting glucose and *GIPR* promoter methylation in DNA from peripheral blood. However, despite accounting for the major confounding factors (age, BMI, diabetes pharmacologic therapy), residual confounding and reverse causation remain possible. We cannot exclude a potential effect of the diet on methylation results in cases. However, there is not published data supporting that a low-carbohydrate diet would affect the methylation pattern of *GIPR* promoter in peripheral blood. We have already controlled for the potential effects of hyperglycemia and antidiabetic medication on the methylation values. A method for overcoming this issue, as proposed by Relton et al [30], is by applying a *“genetical epigenomics”* approach. In our case, this would mean to study the genetic variants that would be related to the methylation pattern, and then to verify whether the correlation with methylation values and insulin resistance remains. However, this was out of scope of the present study.

In conclusion, our research showed that newly diagnosed and drug-naïve T2D patients have impaired β cell function and are more insulin resistant as compared to age- and BMI-matched controls. In addition, adiponectin was lower in T2D patients and correlated with β cell function. IL-12 levels in serum were almost double in T2D patients as compared to controls. The targeted epigenetic analysis in DNA from peripheral blood identified that *GIPR* promoter was hypomethylated in T2D patients as compared to controls. Hypomethylation of *GIPR* promoter correlated with higher fasting glucose and insulin resistance in T2D patients. Further research should unveil the potential role of these findings in the physiopathology of T2D.

## Supporting Information

Table S1 **Peripheral blood DNA methylation values (in %) for each CpG site analyzed in the** ***GIPR*** promoter in type 2 diabetic patients and age- and BMI- matched controls*.
(DOCX)

Appendix S1 **Quantitative DNA methylation analysis.**
(DOCX)
