# Supplementary material for: Gastric Inhibitory Polypeptide Receptor Methylation in Newly Diagnosed, Drug-Naïve Patients with Type 2 Diabetes: A Case-Control Study
Source: PLoS One. 2013 Sep 23;8(9):e75474. doi: 10.1371/journal.pone.0075474 (PMC3781044; doi:10.1371/journal.pone.0075474)
Supplement: Table S1 — Peripheral blood DNA methylation values (in %) for each CpG site analyzed in the GIPR promoter in type 2 diabetic patients and age- and BMI- matched controls*. (DOCX) [file pone.0075474.s001.docx]

## Table S1

**Peripheral blood DNA methylation values (in %) for each CpG site analyzed in the *GIPR* promoter in type 2 diabetic patients and age- and BMI- matched controls*.**

| **CpG site†** | **Position‡** | **Type 2 diabetic patients (n=93)** | **Controls**  **(n=93)** |
| --- | --- | --- | --- |
| CpG 1** | -2126 | 43.6±9.3 | 48.7±9.6 |
| CpG 2*** | -2097 | 63.8±9.7 | 72.9±8.0 |
| CpG 3*** | -2005 | 66.8±6.7 | 63.4±4.2 |
| CpG 4 | -1980 | 3.2±1.1 | 3.6±1.2 |
| CpG 6*** | -1842 | 2.5±0.7 | 4.3±2.1 |
| CpG 7*** | -1833 | 2.5±0.7 | 4.3±2.1 |
| CpG 8** | -1781 | 90.6±7.2 | 94.3±7.1 |
| CpG 9*** | -1526 | 33.7±6.6 | 38.4±8.0 |
| CpG 10*** | -1517 | 33.7±6.6 | 38.4±8.0 |
| CpG 17*** | -1328 | 0.4±0.6 | 2.0±1.0 |
| CpG 18 | -1317 | 1.8±1.2 | 1.6±0.8 |
| CpG 19 | -1295 | 7.6±7.7 | 7.6±3.7 |
| CpG 20 | -1272 | 4.3±0.7 | 4.3±1.2 |

* Values shown are the mean ±SD. P values were calculated using the Mann-Whitney U test. Statistical significance was set at p<(0·05/13) using Bonferroni correction.

*** denotes P value lower than 0·0001.**denotes P value lower than 0·004 (0·05/13).

†CpG dinucleotides have been numbered relative to ATG.

‡CpG dinucleotide position has been determined according to the ATG position for the *GIPR* gene (ENSG00000010310).
