# Supplementary material for: Gastric Inhibitory Polypeptide Receptor Methylation in Newly Diagnosed, Drug-Naïve Patients with Type 2 Diabetes: A Case-Control Study
Source: PLoS One. 2013 Sep 23;8(9):e75474. doi: 10.1371/journal.pone.0075474 (PMC3781044; doi:10.1371/journal.pone.0075474)
Supplement: Appendix S1 — Quantitative DNA methylation analysis. (DOCX) [file pone.0075474.s002.docx]

## Appendix S1:

## Quantitative DNA methylation analysis

Primers used*

| ***GIPR***  **CpGs 1-8 LEFT** | aggaagagagTGGTGTGTGTTTGGAATTTTAGTTA |
| --- | --- |
| ***GIPR***  **CpGs 1-8 RIGHT** | cagtaatacgactcactatagggagaaggctCTCACTCACAAATAAACAATCATCC |
| ***GIPR***  **CpGs 9-20 LEFT** | aggaagagagGTGGATGATTGTTTATTTGTGAGTG |
| ***GIPR***  **CpGs 9-20 RIGHT** | cagtaatacgactcactatagggagaaggctATCACTTACTCCTACAACCCCTACC |

* LEFT: 10-mer tag sequence. RIGHT: T7 promoter tag with an 8 bp insert (for prevention of abortive cycling and constant 5’ fragment for RNaseA reaction).

Capital letters indicate the actual sequence for the primers.

Methylation analysis.

Sequenom's MassARRAY platform was used to perform quantitative DNA methylation analysis. This system utilizes MALDI-TOF mass spectrometry in combination with RNA base-specific cleavage (MassCLEAVE). A detectable pattern is then analyzed for methylation status. PCR primers for the amplification of the promoter of the gene *GIPR* were designed using Epidesigner (Sequenom). When possible, amplicons were designed to cover CpG islands in the same region as the 5′ UTR. For each reverse primer, an additional T7 promoter tag for in vivo transcription was added, as well as a 10-mer tag on the forward primer to adjust for melting-temperature differences. The primers used appear in the above table.

PCR reactions were carried out in 5 μl total volume with 10 ng/ml bisulfite-treated DNA, 0.2 units *Taq*DNA polymerase (Sequenom), 1x supplied *Taq* buffer, and 200 mM PCR primers. Amplification was done as follows: preactivation of 95°C for 15 min, 45 cycles consisting of 95°C for 30 s, 56°C for 30 s, and 72°C for 30 s, finishing with a 72°C incubation for 4 min. Dephosphorylation of unincorporated dNTPs was performed by adding 1.7 ml of H_2_O and 0.3 units of shrimp alkaline phosphatase (Sequenom), incubating at 37°C for 40 min and then at 85º C for 10 min, to deactivate the enzyme. The MassCLEAVE biochemistry was performed as follows: *in vivo* transcription and RNA cleavage was achieved by adding 2 μl of PCR product to 5 μl of transcription/cleavage reaction and incubating at 37°C for 3 h. The transcription/cleavage reaction contains 27 units of T7 RNA&DNA polymerase (Sequenom), 0.64x of T7 R&DNA polymerase buffer, 0.22 μl T Cleavage Mix (Sequenom), 3.14 mM DTT, 3.21 μl H_2_O, and 0.09 mg/ml RNaseA (Sequenom). The reactions were additionally diluted with 20 ml H_2_O and conditioned with 6 mg of CLEAN Resin (Sequenom) for optimal mass-spectra analysis.
